# Extraction Improvement of the Bioactive Blue-Green Pigment “Marennine” from Diatom *Haslea ostrearia*’s Blue Water: A Solid-Phase Method Based on Graphitic Matrices

**DOI:** 10.3390/md18120653

**Published:** 2020-12-18

**Authors:** William Bélanger, Alexandre A. Arnold, François Turcotte, Richard Saint-Louis, Jean-Sébastien Deschênes, Bertrand Genard, Isabelle Marcotte, Réjean Tremblay

**Affiliations:** 1 Institut des Sciences de la mer, Université du Québec à Rimouski, 310 des Ursulines, Rimouski, QC G5L 3A1, Canada; francois.turcotte@dfo-mpo.gc.ca (F.T.); rejean_tremblay@uqar.ca (R.T.); 2 Département de Biologie, Chimie et Géographie, Université du Québec à Rimouski, 300 des Ursulines, Rimouski, QC G5L 3A1, Canada; richard_st-louis@uqar.ca; 3 Department of Chemistry, Université du Québec à Montréal, P.O. Box 8888, Downtown Station, Montreal, QC H3C 3P8, Canada; arnold.alexandre@uqam.ca (A.A.A.); marcotte.isabelle@uqam.ca (I.M.); 4 Département de Mathématiques, Informatique et Génie, Université du Québec à Rimouski, 300 des Ursulines, Rimouski, QC G5L 3A1, Canada; jean-sebastien_deschenes@uqar.ca; 5 Iso-BioKem, 300 des Ursulines, Rimouski, QC G5L 3A1, Canada; bertrand.genard@isobiokem.ca

**Keywords:** graphitized carbon black, graphite, solid-phase extraction, marennine, sulfated polysaccharide, diatom

## Abstract

The compound “marennine” is a blue–green pigment produced by the benthic microalgae *Haslea ostrearia*, with pathogenicity reduction activities against some bacteria and promising potential as a natural pigment in seafood industries. After decades of research, the chemical family of this compound still remains unclear, mainly because structural studies were impaired by the presence of co-extracted compounds in marennine isolates. To improve the purity of marennine extract, we developed a novel extraction method using a graphitic stationary phase, which provides various advantages over the previous procedure using tandem ultrafiltration. Our method is faster, more versatile, provides a better crude yield (66%, compared to 57% for ultrafiltration) and is amenable to upscaling with continuous photobioreactor cultivation. Our goal was to take advantage of the modulable surface properties of the graphitic matrix by optimizing its interactions with marennine. As such, the effects of organic modifiers, pH and reducing agents were studied. With this improvement on marennine purification, we achieved altogether the isolation of a fucoidan-related, sulfated polysaccharide from blue water. Characterization of the polysaccharides fraction suggests that roughly half of UV-absorbing compounds could be isolated from the marennine crude extracts. The identification of sulfated polysaccharides could be a major breakthrough for marennine purification, providing targeted isolation techniques. Likewise, the added value of *Haslea ostrearia* and the role of polysaccharides in previous marennine chemical characterization and bioactivity studies remain to be determined.

## 1. Introduction

Marennine designates a blue–green pigment produced by marine diatom species belonging to the genus *Haslea* [1]. Two distinct forms of marennine are available, one intracellular (IMn), accumulated in the diatom vesicles, and another extracellular (EMn), which is released in the culture medium from the microalgae, and produce the so-called Blue Water (BW) [2]. EMn has been previously studied and showed halochromic proprieties [2], as well as promising prophylactic activity, where marennine does not necessarily act on bacteria growth [3], but rather on pathogenicity factors of some bacteria [4]. More recently, advances in photobioreactor (PBR) cultivation led to the development of a new artificial seawater medium which now enables suspension culture and higher yields through the optimization of calcium, magnesium and iron concentrations [5]. Although nearly two centuries passed since the first marennine studies [1], its molecular structure and chemical family are still not clearly described.

By far, the most extensive characterization to date was published in 2006 by Pouvreau et al. [2]. They assessed different structures for the intra- and extracellular pigment, and proposed a compound related to a polyphenol. While carbohydrates analyses were negative for Pouvreau’s team, study from Gastineau et al. [1] noted NMR signals characteristic of glycosidic elements, suggesting a possible polysaccharide similar to chrysolaminarin. Until now, no experiment has confirmed either of those hypotheses, or the isolation of a pure pigment. Furthermore, an abundance of methyl groups and aliphatic chains were evidenced by NMR analysis [1], which adds to the mystery surrounding the chemical identity of marennine.

In light of the challenges toward the isolation of a pure marennine extract, we developed a novel solid-phase extraction (SPE) method to ease crude extract preparation. The use of carbon media for on-line pre-concentration of polar compounds is promising [6], as stationary phases such as graphitized carbon black (GCB) and porous graphitic carbon (PGC) are highly stable and exert both dispersive and electron-pair donor-acceptor interactions [6,7]. This, in addition to chemically modulable ion-exchange properties [8], allows selective retention of both polar and non-polar analytes. Thus, the unique combination of those intermolecular interactions open new avenues for bulk extraction and purification fine-tuning of marennine on a single stationary phase.

From the chemical information available so far, we expected that marennine has an anionic amphiphilic nature. Hence, to understand its behavior on the graphitic solid phase, we investigated key parameters related to ionic and hydrophobic interactions. As the retention is expected to be mainly driven by those forces, the graphite surface charge, the organic modifier concentration and the pH should exert the strongest effects on pigment recovery. In deference to green chemistry principles, we propose a food-grade method for bulk marennine extraction, by favoring environmentally suitable chemicals whenever possible. Thus, we emphasized the usage of ethanol and ethyl lactate as nontoxic and biodegradable solvents, which can both be produced from renewable feedstocks.

## 2. Material and Method

### 2.1. Algae Culture

Marennine production was conducted at the Station acquicole de Pointe-au-Père (48${}^{\circ }$31′ N; 68${}^{\circ }$28′ W, QC, Canada). Algal production was carried out using an axenic strain of *Haslea ostrearia* (NCC-136) isolated from Bourgneuf Bay, France and provided by Nantes Culture Collection. Cultures were grown in a semi-continuous mode in 50 kDa (KOCH Membrane, Romicon, Wilmington, DE, USA) ultrafiltered seawater enriched with F/2 media [9] and 30 mg·L^−1^ silicates. Batch cultures were produced in 100 L flat bottom circular PBRs at light intensity of 180 μmol photons m^−2^·s^−1^, 14/10 h light/dark cycles, temperature of 20 ${}^{\circ }$C and salinity of 28 ppt. BW containing extracellular marennine from the PBRs was harvested after 35 days, when the concentration reached about 10 mg·L^−1^. BW was filtered at 1 µm with a centrifugal pump to eliminate any cells or particles, and marennine concentration was determined on the cell-free culture water (syringe-filtered on 0.22 μm). Absorbance was measured at the visible _max_ (677 nm) [10], with a cuvette of 10 cm optical path length. Thus, all extracts obtained from different methods came from the same BW production.

### 2.2. Ultrafiltration and Dialysis: Previous Method Overview

Crude purification was conducted as published in Pouvreau et al. [10], with a scaled-up process for the treatment of high BW volumes [1,4]. The ultrafiltration system was composed of a diaphragm pump assuming the feeding of BW through 30 kDa and 3 kDa cutoffs regenerated cellulose spiral membranes (Prep/Scale-TFF cartridges 0.54 m^2^, MilliporeSigma, Oakville, ON, Canada). Circulation flow was adjusted to 6 L·min^−1^ and the working pressure was kept at 25 psi. Marennine was desalted by continuous diafiltration with deionized water, followed by dialysis using a Spectra/Por regenerated cellulose membrane (Repligen, Waltham, MA, USA), with a molecular weight cutoff (MWCO) of 3.5 kDa.

### 2.3. Mobile Phase Optimization

Key parameters of the mobile phase composition were evaluated to optimize marennine recovery extracted from BW, using the SPE-GCB method.

#### 2.3.1. Effect of Organic Modifier

Eluents containing an increasing amount of ethyl lactate were compared. In triplicate, 4% increments of ethyl lactate were used, with concentrations ranging from 18 to 50% (*v*/*v*). Supelco EnviCarb 250 mg cartridges were conditioned with 6 mL ethanol, then 6 mL of an aqueous solution of 60 mM metabisulfite to equilibrate and reduce the stationary phase. Cartridges were loaded with 60 mL BW and washed with 6 mL deionized water. To minimize the hydrolysis of ethyl lactate occurring at alkaline pH, the mobile phase was prepared immediately before the elution of each sample, by mixing the organic solvent with an aqueous buffer (400 mM, ammonium bicarbonate, pH 9) containing 10 mM of sodium sulfite. Marennine concentrate was obtained by eluting 3 mL of the hydroorganic mixture, then analyzed by UV–Vis. Marennine concentration was determined by comparing the absorbance at the λ_max_ of each sample in the visible range, with a cuvette of 1 cm path length.

#### 2.3.2. Effect of Analyte Ionization

The experiment was carried in two parts. First, the onset of the hydroorganic ionization ${}_{w}^{s}$pH was determined through successive elutions, by applying a linear gradient on the mobile phase similarly to the high-performance liquid chromatography (HPLC) method described by Kaliszan et al. for the separation of acids ionogenic analytes [11]. Then, the yield of single extractions was compared among the pH range where the analytes were ionized. The concentration of ethyl lactate was constant for all extractions, at 35% (*v*/*v*) of the final volume, and added immediately prior to elution. Both experiments were conducted in triplicate. Marennine spectra were acquired by UV–Vis and the concentration was determined by comparing the absorbance of each sample at the λ_max_ in the visible range, with a cuvette of 1 cm path length.

Chromophore ionization: Supelco EnviCarb 100 mg cartridges were conditioned with 2.4 mL ethanol, equilibrated with 2.4 mL deionized water, loaded with 24 mL BW, then washed with 2.4 mL deionized water. The charge of the adsorbed marennine molecules was neutralized with 1.2 mL of phosphate buffer (100 mM, pH 2) and eluents containing buffers of increasing pH (100 mM phosphate, ${}_{w}^{w}$pH 2 to 10) were sequentially applied on the same cartridge.

Effective desorption: Eluents with aqueous phase ${}_{w}^{w}$pH ranging from 7 to 10 (${}_{w}^{s}$pH 7.5 to 9.6) were prepared, with incremental steps of 0.5 pH unit. The mobile phase was composed of ethyl lactate mixed with a phosphate buffer (200 mM) containing 10 mM sodium sulfite. Supelco EnviCarb 100 mg cartridges were conditioned with 2.4 mL ethanol, then reduced with 2.4 mL of 60 mM metabisulfite. Cartridges were loaded with 24 mL of BW, washed with 2.4 mL of deionized water, then eluted with 1.2 mL of the hydroorganic mixture. An unbuffered control group was prepared with ethyl lactate and deionized water.

#### 2.3.3. Effect of Ionic Strength

Eluents with increasing buffer concentration were compared. The mobile phase was composed of ethyl lactate (35%, (*v*/*v*)) mixed with a phosphate buffer (60 to 200 mM, pH 8) containing 10 mM sodium sulfite. In triplicate, Supelco EnviCarb 100 mg cartridges were conditioned with 2.4 mL ethanol, then reduced with 2.4 mL of 60 mM metabisulfite. Cartridges were loaded with 24 mL of BW, washed with 2.4 mL of deionized water, then eluted with 1.2 mL of mobile phase. Marennine concentration was determined by UV–Vis, by comparing the absorbance of each sample at the λ_max_ in the visible range, with a cuvette of 1 cm path length.

#### 2.3.4. Effect of Reductants

Eluents with increasing reductant concentration were compared. The mobile phase was composed of ethyl lactate (35%, (*v*/*v*)) mixed with a phosphate buffer (100 mM, pH 8). Sodium sulfite was added in concentrations ranging from 0 to 20 mM. In triplicate, Supelco EnviCarb 100 mg cartridges were conditioned with 2.4 mL ethanol, then reduced with 2.4 mL of 60 mM metabisulfite. Cartridges were loaded with 24 mL BW, washed with 2.4 mL deionized water, then eluted with 1.2 mL of mobile phase. Marennine concentration was determined by UV–Vis, by comparing the absorbance of each sample at the λ_max_ in the visible range, with a cuvette of 1 cm path length.

### 2.4. Stationary Phases Evaluation

#### 2.4.1. Reductants and Repeatability

Two sets of triplicate, with and without reducing agents were compared. Supelco EnviCarb 100 mg cartridges were conditioned with 2.4 mL ethanol, followed by either 2.4 mL of deionized water or sodium metabisulfite 60 mM. The cartridges were loaded with 25 mL BW, washed with 2.4 mL water, then eluted with 1.2 mL of the mobile phase. The eluent was prepared by mixing ethyl lactate (35%, (*v*/*v*)) with a phosphate buffer (100 mM, pH 8), with the addition of 5 mM sodium sulfite for the reductant group. Loading, washing and eluting cycles were repeated 20 times on the same cartridge to demonstrate the method repeatability, while assessing the matrix effect between the stationary phase and marennine. The crude yield of each sample was determined by UV–Vis, by comparing the absorbance of each sample at the λ_max_ in the visible range, with a cuvette of 1 cm path length.

#### 2.4.2. Evaluation of Graphite Flakes As a GCB Alternative

Bulk GCB (EnviCarb, Supelco, Bellefonte, PA, USA) was used as a benchmark for the evaluation of mineral graphite flakes as a low-cost stationary phase alternative. The graphite was wettable flakes, 325 mesh (50–70%), 99+% carbon (Sigma-Aldrich, Oakville, Canada). To determine their saturation point, roughly 500 mg were dried at 105 ${}^{\circ }$C overnight and cooled in a desiccator. In triplicate, 100 mg were weighted on a microbalance, then placed in 50 mL conical centrifuge tubes with 2.4 mL of 60 mM sodium metabisulfite. The tubes were centrifuged 30 min at 4000× *g* and the supernatant discarded. Tubes were filled with 50 mL of BW, mixed and centrifuged again. The partially extracted supernatant was analyzed by UV–Vis and compared against the initial BW concentration to determine marennine retention capacity by weight of the solid phase. Marennine concentration was determined by UV–Vis, by comparing the absorbance of each sample at the λ_max_ in the visible range, with a cuvette of 1 cm path length.

From the results of the previous experiment, the yield by weight of natural graphite flakes was determined by loading the stationary phase with BW at near-saturation. Blank 1 mL SPE cartridges were wet-packed with 100 mg of graphite flakes suspended in ethanol. The graphite was placed between two fritted glass fibers disk and lightly pressed on a vacuum manifold. In duplicate, the cartridges were conditioned with 2.4 mL ethanol, then reduced with 2.4 mL of 60 mM metabisulfite. Cartridges were loaded with 15 mL of BW, washed with 2.4 mL of deionized water and eluted with 1.2 mL of an hydro-organic mixture of ethyl lactate (35%, (*v*/*v*)) and phosphate buffer (100 mM, pH 8) containing 5 mM sodium sulfite. Marennine concentration was determined by UV–Vis, by comparing the absorbance of each sample at the λ_max_ in the visible range, with a cuvette of 1 cm path length. Data from the mobile phase reductants experiment was used for GCB.

### 2.5. Crude Extract Recovery: Antisolvent Precipitation

Dry extracellular marennine was obtained by precipitation of ultrafiltrated BW in a large excess of ethanol (5:1, (*v*/*v*)). The precipitate was collected by centrifugation, dried under a stream of nitrogen, dissolved in an aqueous mixture of ethyl lactate (35%, (*v*/*v*)) and filtered on glass wool. In triplicate, equal amounts of the hydroorganic extract were placed in microcentrifuge tubes, with an increasing concentration of ethanol. Tubes were cooled at 4 ${}^{\circ }$C overnight and centrifuged 15 min at 4 ${}^{\circ }$C, 15,000× *g*. Supernatants were transferred in spectrophotometry cuvettes and gaged at the same volume. Marennine concentration was determined with the absorbance of each sample at the λ_max_ in the visible range, with a cuvette of 1 cm path length. Marennine dissolved in a solution of ethyl lactate (35%, (*v*/*v*)) was used as a control.

### 2.6. Cations Removal

The concentration of Na^+^, K^+^, Ca^2+^ and Mg^2+^ was first determined in BW, then at each key steps of the crude extract preparation; after precipitation, precipitate wash, and after treatment with a strong cation-exchange resin (SCX). In triplicate, three sets of samples were prepared to measure cations concentration at each stage. Crude extract was used from the repeatability experiment with reductants. A volume of 3 mL of hydroorganic extract was transferred into 15 mL conical centrifuge tubes, then mixed with ethanol to induce precipitation (2.5:1, (*v*/*v*)). Samples were cooled at 4 ${}^{\circ }$C overnight and centrifuged for 20 min at 4200× *g*, 4 ${}^{\circ }$C. Supernatant was discarded, and one set was solubilized in 3 mL nanopure water, then prepared for cation analysis. Precipitates of the two remaining sets were washed with 10 mL of ethanol solution (80%, (*v*/*v*)) pre-chilled at −20 ${}^{\circ }$C, then centrifuged again. The pellets were solubilised in 3 mL nanopure water, and one sample set was purified on a SPE-SCX cartridge (100 mg, sulphonic acid, Grace/Alltech, Columbia, SC, USA). The resin was conditioned with 3 mL ethanol and 3 mL deionized water prior to sample elution. Each set was analyzed by UV–Vis, then acidified with nitric acid (HNO_3_, 3.65%, (*v*/*v*)) and syringe-filtered on polytetrafluoroethylene (PTFE) filters (13 mm, 0.45 μm, VWR, Edmonton, Canada). Samples were analyzed with a microwave plasma-atomic emission spectrometer (MP-AES), where cations abundance was calculated from a six-points rational calibration curve, with concentration ranging from 0.05 to 20 mg/L. The nitric acid used was Instra-analyzed, and all glassware was acid-washed with dilute HNO_3_ (10%, (*v*/*v*)). Samples were analyzed as is for K^+^, Ca^2+^ and Mg^2+^, and diluted 1:1000 for Na^+^. Blue water was diluted by 1:50 for K^+^, Ca^2+^ and Mg^2+^, and 1:10,000 for Na^+^.

### 2.7. Purification

In triplicate, Supelco EnviCarb 250 mg cartridges were conditioned with 6 mL ethanol, followed by 6 mL sodium metabisulfite 60 mM. The cartridges were loaded with 60 mL BW then washed with 6 mL water. Water was removed with 6 mL ethanol and the polysaccharides fraction was recovered with 3 mL of a dichloromethane (DCM) and methanol (MeOH) mixture (6:4, (*v*/*v*)) containing 0.2% (*v*/*v*) trifluoroacetic acid (TFA). Residual DCM was removed with 6 mL ethanol, and the adsorbed marennine molecules were ionized with 3 mL phosphate buffer solution (100 mM, pH 8). The marennine fraction was eluted with 3 mL of a mixture of acetonitrile (35%, (*v*/*v*)) and phosphate buffer (100 mM, pH 8) containing 5 mM sodium sulfite. Acetonitrile was used in replacement of ethyl lactate for its low UV cutoff wavelength.

The polysaccharides fractions were dried under a nitrogen stream and resolubilized in 3 mL methanol for UV–Vis analysis. The samples were transferred into glass vials, dried and weighted on a microbalance. The extracts were dissolved in 15 μL methanol, then dried on an attenuated total reflection (ATR) crystal and analyzed by Fourier-transform infrared spectroscopy (FT-IR). Another extraction was performed in the same manner, in triplicate, where the analytes were resolubilized in 3 mL water instead of methanol to study their absorbance without solvent cutoff artifacts.

A sulfated polysaccharides sample was prepared for NMR following the same purification procedure, scaled up 20 times. Two fritted glass fibers disks were added into a 60 mL syringe, wet-packed with 5 g of GCB (EnviCarb, Supelco, Bellefonte, PA, USA) suspension in ethanol. The crude extract was dried with a rotary evaporator and dissolved into 500 μL of deuterated dimethyl sulfoxide (DMSO-d6). Another sample was prepared in the same manner and deacetylated by alkali treatment, as described in Tako et al. [12]. Briefly, the sample was stirred at 24 ${}^{\circ }$C for 14 h in 5 mL of sodium hydroxide 50 mM and sodium chloride 13.5 mM under nitrogen atmosphere. The sample was neutralized with HCl, filtrated on Celite 545, dialyzed in a regenerated cellulose membrane of 3.5 kDa MWCO (Spectra/Por) and freeze-dried.

The marennine fractions were transferred into 15 mL conical centrifuge tubes, then precipitated with ethanol (2.5:1, (*v*/*v*)). Samples were cooled at 4 ${}^{\circ }$C overnight and centrifuged during 20 min at 4200× *g*, 4 ${}^{\circ }$C. Supernatant was discarded, and the precipitate washed with 10 mL ethanol solution (80%, (*v*/*v*)) pre-chilled at −20 ${}^{\circ }$C. Samples were centrifuged again and the precipitate resolubilized in 3 mL of deionized water for UV–Vis analysis. Absorbance was measured in a cuvette of 1 cm path length.

### 2.8. Instruments

UV–Visible (UV–Vis) spectra were acquired on a spectrophotometer (Cary 100, Agilent/Varian, Santa Clara, CA, USA), using Varian WinUV software (version 3.00). Samples for UV–Vis were analyzed at 24 ${}^{\circ }$C, in quartz cuvettes. Other instruments used were the microbalance Cubis 3.6P-2500-M (Sartorius, Goettingen, Germany), freeze dryer FreeZone 2.5 (Labconco, Kansas City, MO, USA), microcentrifuge PrismR and the centrifuge Sigma 3-18KS with rotor 11,180 (MBI Lab Equipment, Dorval, QC, Canada). pH values were recorded on an Accumet basic AB15 with a standard pH combination electrode 13-620-287a (Thermo Fisher Scientific, Waltham, MA, USA). ATR-FTIR spectra were acquired on a Nicolet 6700 (Thermo Fisher Scientific), between 400 and 4000 cm^−1^, with 64 scans per sample and a resolution of 2 cm^−1^, using OMNIC software (7.3). Cations determination was carried with a MP-AES (4200, Agilent, Santa Clara, CA, USA), using MP Expert software (1.6.0.9255).

NMR samples were analyzed using a Bruker Avance III HD spectrometer operating at a ^1^H frequency of 599.9 MHz using a double-resonance 5 mm BBFO probehead. The proton spectra were recorded with a single 30${}^{\circ }$ pulse, 16 repetitions and an acquisition time of 2.73 s. Residual moisture signal was eliminated by pre-saturating the water peak during the 2 s recycle delay. ^1^H-^13^C heteronuclear single quantum correlation (HSQC) spectra with multiplicity editing were acquired with 48 repetitions and 324 increments, applying the echo-antiecho scheme with an acquisition time of 166 ms. Data was analyzed using Bruker TopSpin software (4.0.6).

### 2.9. Statistical Analysis

Data are expressed as means (±standard deviation). Normality was tested by a Shapiro–Wilk test and the homoscedasticity was assessed with Leneve’s test. When the null hypothesis of a normal distribution could not be rejected for a set of samples, the difference between treatments was validated with an analysis of variance (ANOVA), followed by a post hoc Tukey HSD test. Else, the difference was validated with a Kruskal-Wallis test, followed by a Wilcoxon rank-sum test using normal approximation without continuity correction. Data were analyzed using the R software (version 4.0.2-1), with packages car (3.0.7), agricolae (1.3.2), multcompView (0.1.8) and rcompanion (2.3.25). For all statistical analysis, an alpha value of 0.05 was considered significant.

## 3. Results

### 3.1. Ultrafiltration Crude Yield: Previous Method

The yield using the previous ultrafiltration method was determined according to Beer-Lambert law, by using the molecular mass assessed by Pouvreau et al. for EMn (9893 ± 1 Da) [2]. Thus, the yield of marennine crude extract after ultrafiltration between 3 and 30 kDa was estimated at 56.6 (2.6)%. The lower value compared to the reference crude yield is likely due to the scale-up of the ultrafiltration procedure and the usage of a different strain of *Haslea ostrearia* (NCC-136), comparatively to Pouvreau et al. [10]. BW concentration and ultrafiltration yields are detailed in Table 1.

### 3.2. Overview of the Novel SPE-GCB Method

**Figure 1:**
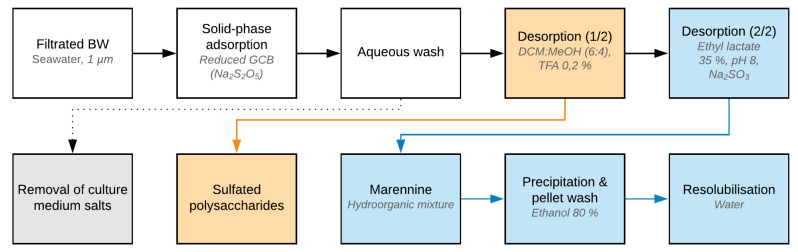
Schematic representation of the optimized solid-phase extraction (SPE)-graphitized carbon black (GCB) extraction process of extracellular marennine harvested from BW. Concentrations are expressed as (*v*/*v*).

The main steps and parameters of the optimized SPE-GCB procedure are summarized in Figure 1, including an optional purification step for the recovery of a sulfated polysaccharides fraction.

### 3.3. Effect of Organic Modifier

**Figure 2:**
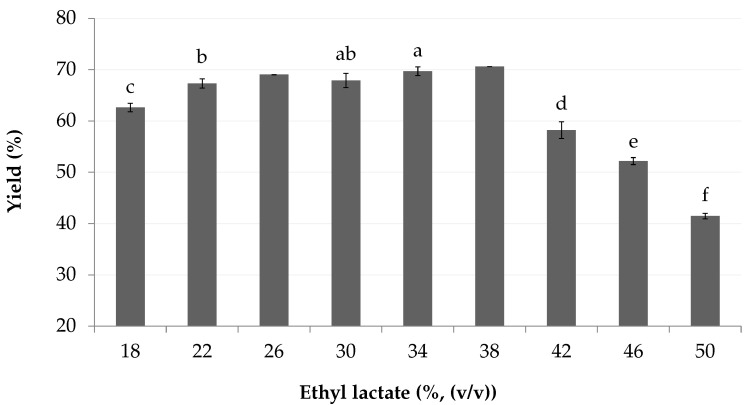
Crude extraction yield according to ethyl lactate concentration in the mobile phase. As values at 26 and 38% ethyl lactate (*v*/*v*) are unbalanced, containing only two samples instead of three, statistical differences with other concentrations could not be calculated adequately.

The extraction yield was improved up to 70% as the concentration of ethyl lactate increased (Figure 2), where concentrations above 38% (*v*/*v*) showed a linear decrease of yield. Thus, the recommended proportion of ethyl lactate to use is between 34 and 38% (*v*/*v*).

### 3.4. Effect of Analyte Ionization

**Figure 3:**
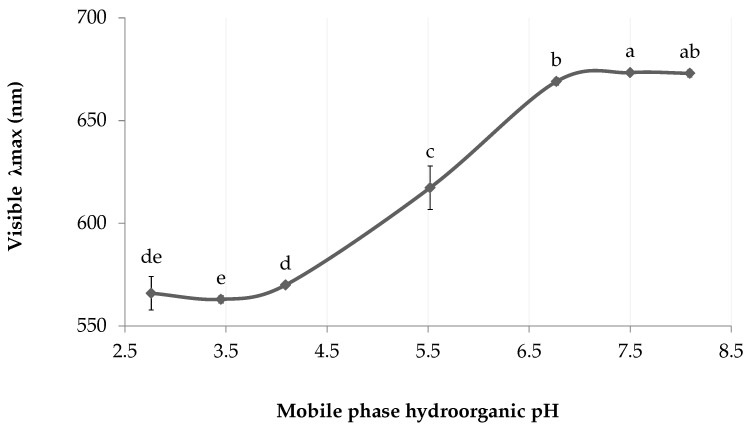
Approximation of the hydroorganic dissociation constant (${}_{w}^{s}$pK_a_) of EMn in ethyl lactate solution (35%, (*v*/*v*)). Letters indicate statistical differences between treatments.

In the chromophore ionization experiment (Figure 3), the visible λ_max_ undergoes a bathochromic shift as the pH increases. Similarly to the method described in Berkhout et al. for spectrophotometric pK_a_ determination [13], the maximum absorption wavelengths were plotted against the mobile phase ${}_{w}^{s}$pH values, which translates into a sigmoid shaped curve. The inflexion point (5.5) gives an approximation of the chromophore dissociation constant (pK_a_), where half of the chromophores are ionized. Thus, marennine chromophore appears fully ionized from ${}_{w}^{s}$pH 6.8 and above.

**Figure 4:**
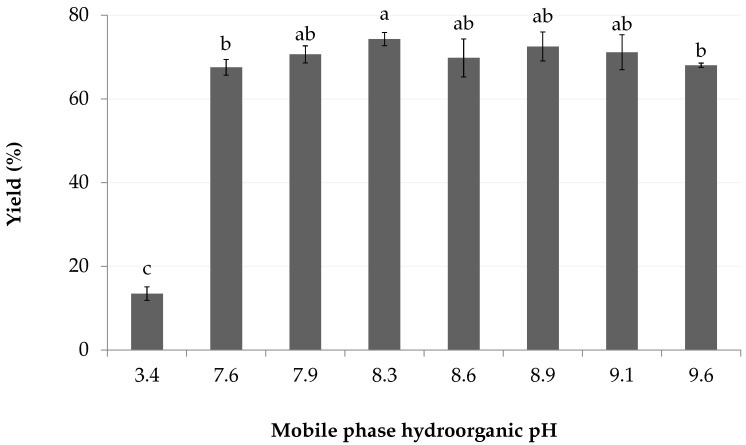
Effect of mobile phase ${}_{w}^{s}$pH on the crude yield. Samples are buffered with 200 mM phosphate, control is unbuffered (3.4). Letters indicate statistical differences between treatments.

In accordance with the approximation of marennine ${}_{w}^{s}$pK_a_ in dilute ethyl lactate, the extraction yields obtained with mobile phase of ${}_{w}^{s}$pH values between 7.9 and 9.1 were not significatively different, where slight differences for the higher (9.6) and lower ends (7.6) are assumed to result from experimental variance (Figure 4). The average yield for pH 7.6 and above was 71 (2)%, in contrast to the unbuffered control which is drastically lower, at 13 (2)%, being acidified by the organic solvent.

### 3.5. Effect of Ionic Strength

**Figure 5:**
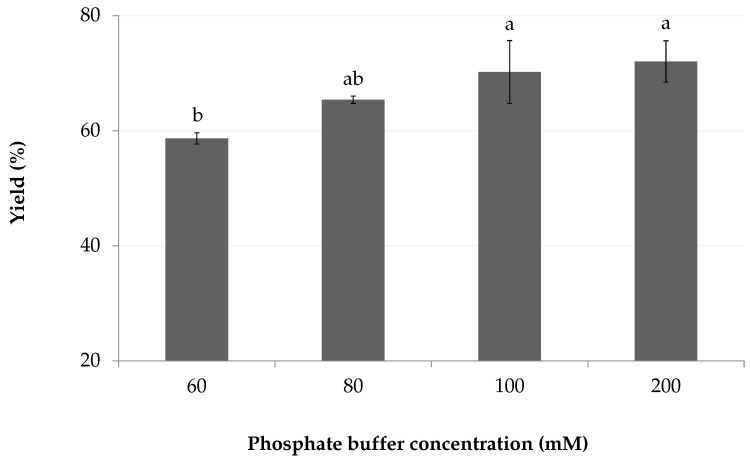
Effect of mobile phase ionic strength on crude yield. Increase of buffer concentration with constant pH and mobile phase composition. Letters indicate statistical differences between treatments.

**Figure 6:**
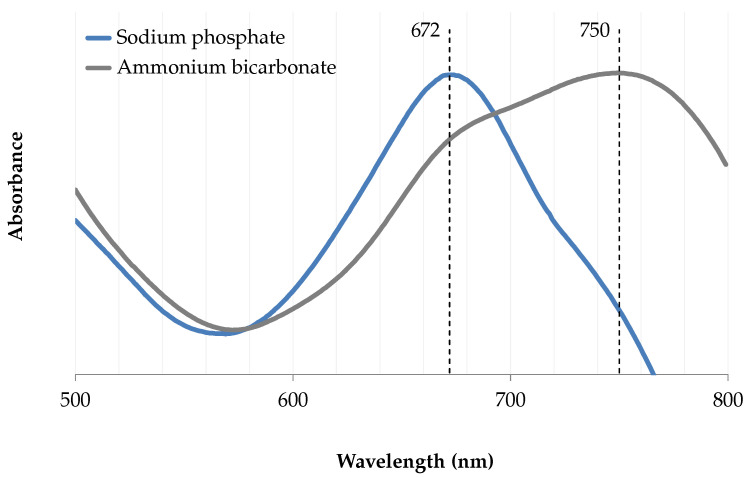
Bathochrome shift of the visible absorption band in ammonium bicarbonate (400 mM), compared to sodium phosphate buffer (200 mM).

An increase in ionic strength from 60 to 200 mM slightly improved the crude yield (Figure 5), from 59 (1)% to 71 (4)% (100–200 mM). The lowest buffer concentration (60 mM) allowed a constant ${}_{w}^{s}$pH of 8 following the addition of ethyl lactate. A high concentration of some buffers, such as ammonium bicarbonate, caused an adverse reaction where marennine color changed from green to brown, with a pronounced absorbance bathochrome in the visible region. The λ_max_ moved to 750 nm, compared to 672 nm with a phosphate buffer (Figure 6).

### 3.6. Effect of Reductants

**Figure 7:**
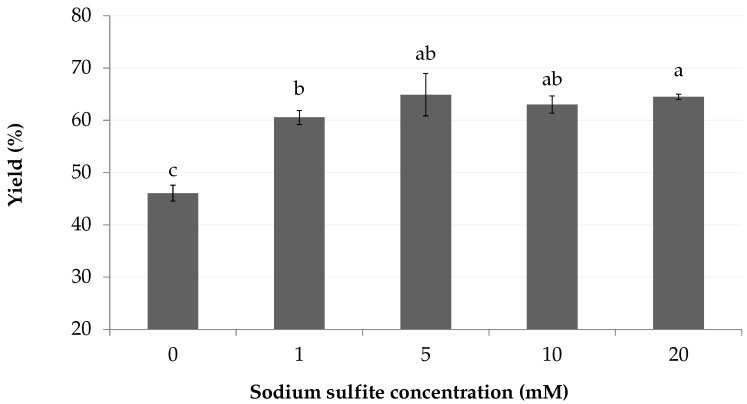
Effect of reductant (sulfite) concentration of mobile phase on the crude yield. The stationary phase was pretreated with sodium metabisulfite (60 mM). Letters indicate statistical differences between treatments.

The addition of sodium sulfite in the mobile phase increased the yield by roughly one third, from 46 (2)% to 63 (2)% (Figure 7). The yields between 5 and 20 mM were not significatively different, which suggests that a low reductant concentration is sufficient to induce desorption.

### 3.7. Stationary Phases Evaluation

The stationary phase was reduced with sodium metabisulfite to improve the recovery of anionic analytes. Likewise, sodium sulfite was added in the mobile phase. Sodium sulfite was preferred over dithionite or metabisulfite in the eluant, as it effectively improves marennine recovery while being weak enough to avoid reduction of the pigment (not shown).

#### 3.7.1. Reductants and Repeatability

**Figure 8:**
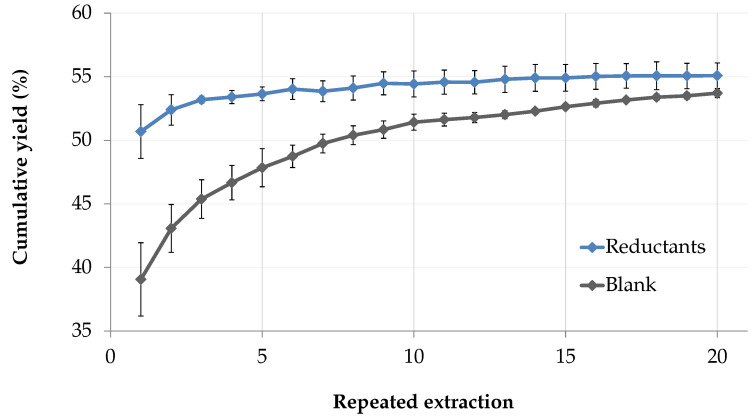
Matrix effect evaluation following stationary phase pretreatment with sodium metabisulfite (60 mM) and addition of sodium sulfite (5 mM) in the mobile phase. Comparison of the average cumulative yield with and without reductants after 20 successive extractions.

The yield of the first extraction (Table 2) was greatly increased with reductants, indicating ionic interactions between the solid phase and the marennine molecules. While the effect was pronounced for the first fractions, it quickly decreased as the extractions were repeated. The cumulative yield of extractions 1 to 20 shows a logarithmic growth (R^2^ = 0.9898) for the control group, while the matrix effect is mostly canceled with reducing agents (Figure 8).

#### 3.7.2. Evaluation of Graphite Flakes As GCB Alternative

Determination of the saturation point of each stationary phase has shown that while GCB had the best retention capability, the crude yield at near-saturation was not significantly different between GCB and natural graphite flakes (Table 3). Thus, mineral graphite flakes appear to be a strong contender, reaching 68% of GCB retention.

### 3.8. Crude Extract Recovery: Antisolvent Precipitation

**Figure 9:**
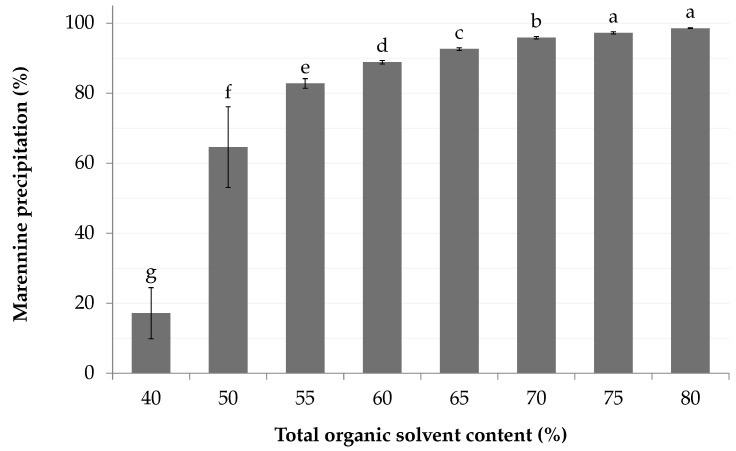
Precipitation yield according to total organic solvent concentration, with addition of ethanol to a marennine solution containing ethyl lactate (35%, (*v*/*v*)). Letters indicate statistical differences between treatments.

Marennine solubility has readily decreased as the concentration of organic solvents increased (Figure 9). The recovery through precipitation reached 99% with the addition of 2.4 mL of ethanol per milliliter of the mobile phase. At this concentration, the organic solvent concentration accounts for 80% of the sample volume. The marennine sample is deemed as a precipitable fraction, as it was prior extracted by ethanol precipitation. Thus, the true yield might be lower.

### 3.9. Cations Removal

The concentration of cations others than Na^+^ was greatly reduced at the precipitation step. Acknowledging the volumetric concentration factor, for which 62.5 mL of BW was concentrated into 3 mL of hydroorganic extract, the abundance of magnesium ions dropped by more than three thousand folds relative to the concentration found in BW. Therefore, the aqueous washing step during the SPE-GCB appears highly effective to remove most salts. Thus, the high concentration of leftover sodium ions likely arise from the sodium phosphate buffer, which is only partially eliminated at the precipitation step. Similarly, pellet rinse with ethanolic solution had little effect on cation abundance. In contrast, SPE-SCX removed nearly all traces of cations, although anions were left behind. Results from the cation determination are shown in Table 4.

### 3.10. Purification

Prior to marennine elution, an additional washing step achieved the isolation of a brown-tinted component. The crude yield of the methanol-soluble fraction was 12.8 (0.2) mg per liter of blue water. Marennine mass could not be determined precisely due to leftover buffer counterions.

#### 3.10.1. ATR-FTIR Analysis

**Figure 10:**
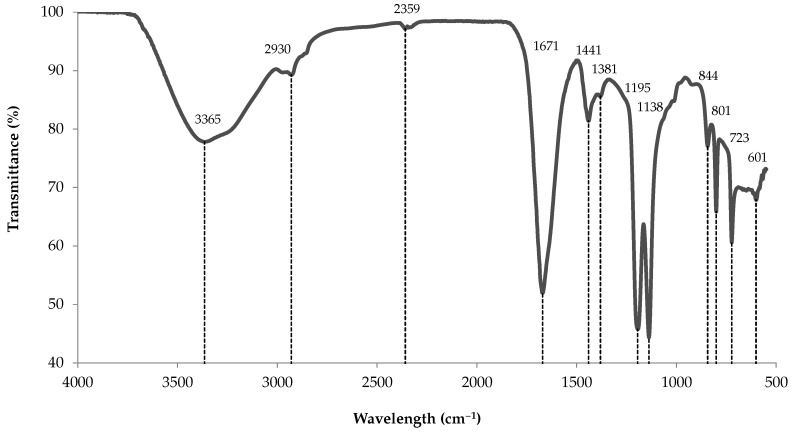
Attenuated total reflection (ATR)-FTIR spectrum of sulfated polysaccharides extracted from blue water.

The infrared spectrum of the methanol-soluble fraction (Figure 10) share the same bands as a fucoidan extract from the brown algae *Padina tetrastromatica* (Table 5). While the fingerprint region strongly matches *P. tetrastromatica* spectrum, there is a slight shift of the O–H broad stretching band, with the presence of two additional weak bands at 2930 and 2359 cm^−1^. Still, those two bands were also found in a commercial fucoidan extract from *Ascophyllum nodosum* (Ascophyscient^®^), as well as in a further purified version following precipitation of polyuronic acids residues (i.e., ascophyllan) with calcium acetate [14]. To our knowledge, assignment of the 2359 cm^−1^ band could not be found in the literature for fucoidans, but was assigned to O–H vibration of carbohydrates in bacterial extracellular polymeric substance [15].

The experimental spectrum comprises all bands typical of polysaccharides, including a strong and broad O–H stretching at 3500–3000, C–H stretching at 3000–2800, O–C–O asymmetric stretching at 1630–1600 and 1400 (here 41 cm^−1^ higher), with a weak C–H bending near 1380 cm^−1^ [18]. In a fucoidan extract from *Sargassum henslowianum*, the band around 1400–1470 cm^−1^ was also ascribed to methylene (CH_2_) scissoring vibration in the case of mannose and galactose, and to methyl (CH_3_) asymmetric bending in fucose and *O*-acetyl [19]. The spectrum also possesses the three bands that are characteristic of fucoidan compounds in *Sargassum oligocystum* and *P. tetrastromatica* species, near 1437, 1203 and 1140 cm^−1^ [16]. Likewise, the fingerprint region contains all the peaks attributed to sulfur-bonded atoms, from 1142 to 723 cm^−1^ [16]. Of those, the two bands near 801 and 842 cm^−1^ were previously ascribed to sulfated carbohydrates in carrageenans, where 845 cm^−1^ was assigned to *D*-galactose-4-sulfate [20]. For fucoidans, they were assigned to sulfation in axial and equatorial positions in *P. tetrastromatica*, respectively, and 844 cm^−1^ to axial C-4 position on fucopyranose residues in *Fucus serratus* [16,21].

#### 3.10.2. UV–Vis Analysis

**Figure 11:**
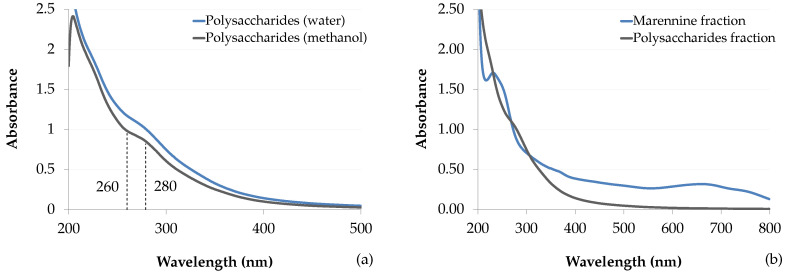
(**a**) UV–Vis spectra of the isolated polysaccharides solubilized in water and methanol. (**b**) UV–Vis spectra of the two fractions obtained from the SPE-GCB method. Fractions were resolubilized in water at their original volume.

From the UV–Vis spectra of the methanol-soluble fraction, the purity of various marennine preparations was evaluated by comparing their absorbance at wavelength ratios which are characteristic of the marennine chromophores. In accordance with the previously published molar attenuation coefficient for EMn [2], wavelengths of 247, 322 and 677 nm were used. The UV–Vis spectrum of the polysaccharides extract (Figure 11a) overlaps the UV region of purified marennine (Figure 11b), and shows a great resemblance to IMn spectrum, which has no peak at 322 nm [10], but rather a small shoulder much alike the extracted polysaccharides. The spectrum also closely matches the one obtained from *Sargassum vachellianum* fucoidan-rich polysaccharide extract, with a sharp peak in the UVC region, a shoulder near 280 nm and no absorbance in the visible spectrum [22]. The light bathochrome seen in *S. vachellianum* extract most likely results from different solute concentrations.

From the overlap between the spectra of marennine and sulfated polysaccharides, wavelength ratios were used as a proxy for the determination of UV-absorbing components in marennine samples (Table 6). Sulfated polysaccharides and all marennine extracts shared a similar ratio for UV wavelengths, at 247/322 nm. In contrast, the UV-to-visible ratio (247/677 nm) of the anion-exchange purified sample is lower than both crude extracts. The apparent stability of the UV ratio, regardless of the preparation method, suggests that the absorbance in the UV region mainly stems from compounds unrelated to marennine chromophores. Hence, those results could indicate similar purity between the ultrafiltrated and the SPE-GCB crude extracts. Following reconstitution, the UV–Vis spectra show similar absorbance in the UV region of marennine and the isolate (Figure 11b), suggesting that the contamination from UV-absorbing compounds was reduced by half.

#### 3.10.3. NMR Analysis

Following the scaled-up extraction, 15 mg of the methanol-soluble fraction was recovered for NMR analysis. The ^1^H-^13^C HSQC and ^1^H spectra (Figures S1 and S2) share some similarities with the latest published spectrum of marennine [1], with ^13^C/^1^H resonances at 10–25/0.90 ppm (CH_3_), including an intense crosspeak at 28/1.22 ppm in marennine and 28/1.24 ppm in our sample (CH_2_). Both spectra are dominated by signals from the ring carbons (60–75/3.5–4.5 ppm) and the aliphatic regions (10–40/1.0–2.5 ppm). This is in agreement with some spectral features observed with fucoidan from brown algae for which ^1^H signals at 1.1–1.3 and 1.24 ppm were attributed to methyl protons of *L*-fucopyranose moieties [12,19]. Moreover, resonances at 60–85 ppm were associated with fucoidan ring carbons [12], among which we found signals at 65–69/3.2–4.1 ppm, previously ascribed to CH_2_ of non-fucose residues, such as xylose and galactose [23].

However, in contrast to both marennine and fucoidan spectra, no anomeric carbons were found at 95–105/4.3–5.8 ppm nor 95–105/5.0–5.5 ppm in our methanol-soluble sample [12,19,23]. Although signals may be masked due to random sulfation and acetylation of the carbohydrates [24,25], the absence of characteristics resonances near 100 ppm refutes the presence of fucose, glucose and xylose, and thus contradicts the prospect of fucoidan. Instead, the spectrum shows high-field anomeric signals at 110/4.7 ppm and 113/4.9 ppm, most likely attributable to arabinose. Although the latter is common in brown seaweeds and diatoms mucilage, it is usually present in low proportions [19,26,27], while a major arabinose content is more typical of green algae polysaccharides, among which sulfated arabinan containing up to 57% arabinose was reported [28,29,30].

In its native state, the compound was insoluble in DCM and soluble in water, methanol and DMSO. Although the analysis of the deacetylated sample was inconclusive, its solubility in DMSO successfully decreased, and the retention during dialysis confirms its polymeric nature.

## 4. Discussion

### 4.1. Optimization of Extraction Parameters

Our results showed that the solid-phase extraction with carbonaceous media is a successful method for pre-concentration of the blue pigment marennine, respecting green chemistry principles and providing a crude yield near 70%. Regarding the various intermolecular interactions in the graphitic stationary phase, we evaluated several parameters to optimize the composition of the mobile phase and improve marennine recovery. As expected for carbonaceous sorbents, the mobile phase pH, organic modifier concentration and the stationary phase surface charge all exerted a significant effect on marennine crude yield. We found evidence of both ionic and dispersive interactions, and following the use of reducing agents, marennine desorption was mainly driven by a typical chromatographic reversed-phase behavior. Hence, the yield depends on the analyte state of ionization, its solubility, and the eluting strength of the mobile phase.

Results have shown that marennine is more effectively desorbed at alkaline pH. This is in agreement with the studied behavior of ionogenic solutes on carbonaceous sorbents, as neutral compounds are more strongly retained on the stationary phase, showing similarity to reversed-phase chromatography [31,32]. The chromophore ionization experiment indicated an apparent ${}_{w}^{s}$pK_a_ of roughly 5.5 for EMn in 35% ethyl lactate, which is 1.5 higher than the ${}_{w}^{w}$pK_a_ previously reported (4.02 ± 0.02) [2]. This most likely results from the influence of the organic solvent on pH values, which is expected to differ from the standard reference [11,33]. As *Haslea ostrearia* is grown in seawater, a mobile phase of pH 8 was chosen. Thus, the solution pH in which marennine is dissolved remains constant through cultivation and extraction, while providing chromophore ionization and sulfites stability [34].

Similar to the use of acetonitrile on PGC [35], the positive relationship between increasing organic modifier concentration and desorption is assumed to result from the competition with dispersive interactions on the graphitic surface. Their strength could result from the planarity and the polarisable surface of graphitized sorbents, which might favor the contact with analytes and induce those forces [36]. Hence, previous studies described a similar reversed-phase behavior for chromatographic separation of purines and opiates on PGC [31,32]. Barret et al. also noted that compounds with acidic functional groups are mostly affected [32], which is in line with the low dissociation constant of EMn (4.02 ± 0.02) [2]. In contrast, the yield decreased as the concentration of ethyl lactate rose above 38%, corresponding to the results from the crude extract recovery experiment where precipitation began at the lowest concentration tested (40% of total organic solvent volume). Thus, a concentration of 35% was chosen in order to provide satisfactory desorption and ensure solubility of marennine in the mobile phase. High concentrations of pH buffers were used to provide a stable pH despite the varying ethyl lactate concentration, as this solvent undergoes a base-catalyzed hydrolysis at alkaline pH [37], which releases lactic acid and thus lowers the pH of the mobile phase over time.

As the choice of solvents deeply defines the environmental performance of a process [38], efforts were made to find a greener alternative to acetonitrile early in the method development. Water, alcohols, ethers and esters are usually preferred [38,39], hence in extraction processes a promising avenue is the utilization of bio-based solvents in replacement of those derived from petrochemistry [39]. Moreover, as they are more likely to benefit from a decentralized production and a shorter supply chain [40,41], green alternatives may further reduce energetic costs associated with transportation, while increasing resilience to global shortages. Ethyl lactate is regarded as a promising candidate as it can be produced from carbohydrate feedstocks, is biodegradable, easy and inexpensive to recycle, readily metabolized in vivo, and has a very low ecotoxicity [37,42]. Ethyl lactate has a high boiling point and is a polar protic solvent, with effectiveness comparable to traditional solvents such as acetonitrile [42,43]. Lastly, Hennion reduced solvent usage by 67% by reversing the extraction cartridge prior to elution, and recommends always using this method for carbonaceous sorbents. While using less solvent, back-flush desorption could also improve marennine yield as it has shown improved recovery for more than half of the analytes tested [44].

Increasing the ionic strength had a moderate effect on crude yield, in agreement with a previously reported separation of charged compounds on graphitized carbon [45]. However, the high buffer concentration requires additional steps for desalting, which may hinder those gains. Although the use of ethyl lactate is advantageous from an environmental perspective, an aprotic solvent such as acetone might be more suited for marennine extraction, requiring minimal buffering. Acetone is considered a greener alternative to acetonitrile, and has similar physicochemical properties and separation performance in reversed-phase HPLC [39,46]. Moreover, acetone has a low boiling point (56 ${}^{\circ }$C) and does not form an azeotrope with water, thus it can be easily recovered by distillation [47]. Although ethyl lactate was favored from a safety perspective, for its high boiling point and low vapor pressure, there might be an interesting tradeoff with repeated extraction using acetone as the organic modifier.

Carbonaceous sorbents contain positively charged groups such as oxonium and quinones, which act as anion-exchange sites [44]. Negatively-charged compounds may bind too strongly on those sites, and pretreatment with ascorbic acid in dilute hydrochloric acid (HCl) can improve their recovery by altering the surface charge of the stationary phase [8,44]. As an alkaline counterpart, sodium metabisulfite was used in replacement of ascorbic acid and HCl. Thus, the stationary phase was pretreated with metabisulfite, leaving an apparent negative charge at the surface of the GCB. When marennine extraction was repeated multiple times on the same stationary phase, the yield of the first fractions was much higher with reductants, both as sorbent pretreatment and mobile phase additive. Theses results indicate the presence of ionic interactions between GCB and the solute, which can be chemically mitigated. As the yield reaches a plateau, this suggests that GCB undergoes a progressive saturation of its positively charged groups. This is supported by numerous studies reporting a chromatographic behavior resembling a weak anion-exchange, with negligible cation-exchange capacity [36]. In its native state, it has been speculated that PGC has an oxidizing activity, which can be altered by redox treatments [36]. Oxidation seems to increase the positive surface charge, thus causing a stronger retention of anions, while reduction decreased anionic interactions and may improve solute recovery [8,36,48]. Hence, redox treatments strongly influence ionogenic compounds, which fits the description of marennine being a polyanionic species [2,36].

Likewise, the addition of sodium sulfite in the mobile phase has a pronounced effect on yield. It has been reported that its use in the mobile phase acts as a redox buffer that cancels a catalytic reaction between dissolved oxygen and PGC surface, thus preventing oxidation of analytes and decreasing the retention of anionic compounds [36,49]. It has been shown that upon passage through a PGC column, the sulfite ions are completely converted to sulfates, suggesting that the oxidation of carbonaceous sorbent might be driven by the concentration of oxygen in the mobile phase [50]. Thus, the use of redox buffers and sorbent pretreatment is advisable to improve reproducibility in chromatographic separations [8].

Natural graphite flakes seem to be a promising alternative for the low-cost pre-concentration of marennine. From our results, they provide similar performance to GCB, at a fraction of the cost. Graphite retails at about $0.04 per gram (USD), i.e., over 200 times cheaper than bulk GCB. Moreover, the retention capacity of graphite could be further improved by chemical exfoliation to produce expanded graphite, which has a greatly increased surface area [51].

Results from the precipitation experiment have shown that ethanol is a suitable anti-solvent for marennine. While it induces precipitation, the use of volatile solvents such as ethanol is mandatory for the removal of residual ethyl lactate in the crude extract, as they have an excellent miscibility [37] and ethanol has a much lower boiling point (78 ${}^{\circ }$C) [52]. As a chemical precursor and degradation product of ethyl lactate [37], ethanol also prevents the production of complex solvent wastes. Pouvreau has shown that contaminating pigments, such as chlorophylls and carotenoids were eliminated by precipitation with acetone [53]. We assume that those would also remain in the ethanolic supernatant, along with the methanol-soluble fraction.

Cations concentration was monitored through the precipitation and pellet washing steps. Our results showed that most ions from seawater were removed during the aqueous wash of the SPE-GCB procedure, but that the sodium ions from the pH buffer remained in the final extract. Solid–liquid extraction of the marennine pellet had little effect on the concentration of cations, either due to poor solubility in the ethanol solution, bonds between cations and the polyanionic structure, or limited contact. As the pellet is repulsed by the organic solvent, it is tightly held together even after prolonged mixing. In order to improve purification, we suggest that ultrasound-assisted extraction might help to disrupt the precipitate. In contrast, residual cations were nearly eliminated after elution on a cationic exchange resin (SPE-SCX). It appears that SCX decreased the extraction yield, although there was no apparent retention of marennine. This might result from loss during the manipulation of small sample volume rather than unrecoverable loss due to adsorption. However, the resulting acidification might be detrimental to marennine structural integrity. Due to a large amount of residual ions, dialysis remains more appropriate as a gentler desalting step. Salt concentration could also be minimized by using a volatile buffer, but sodium phosphate was preferred to avoid an adverse reaction observed with ammonium bicarbonate.

### 4.2. Extraction and Characterization of a Sulfated Polysaccharide

In their review on PGC, West et al. reported that some molecules exert strong dispersive interactions with the carbon surface, requiring solvents with high eluting strength [36]. Likewise, Hennion used a mobile phase composed of dichloromethane and methanol to improve pesticides desorption, with the addition of TFA for acidic compounds [44]. We used a variation of this method to extract a component from BW, which was further characterized. Results from both UV–Vis and FTIR analyses match those of fucoidan references. For the latter, the functional groups region bears all the expected bands for polysaccharides, along with fingerprint signals which correspond to all observed bands in fucoidan from the brown algae *P. tetrastromatica*. Among those, the fingerprint includes signals stemming from sulfur-bonded carbohydrates, which are typical of marine sulfated polysaccharides.

Fucoidans are complex, diverse, high molecular weight sulfated polysaccharides, composed of a fucose backbone (homofucans) or possessing fucose side branches (heterofucans) [24]. They contain a variable amount of monosaccharides and uronic acid, with random branching, sulfation and acetylation, sometimes even with protein moieties [54,55]. They are typically found in brown algae [56], but also produced by sea cucumbers [54] and recently identified in diatoms [24]. In diatoms, they were observed when excreted as exopolysaccharide (EPS) similar to fucoidan, although their structure remains uncharacterized and might differ from brown algae extracts [24]. Analysis of fucoidan from *Chaetoceros socialis* revealed a rather low fucose content (10%) compared to brown algae (17–44%), being the least abundant monosaccharide after glucose, xylose and galactose [24]. As EPS, they act as a surfactant and provide a polyanionic nucleus for the formation of marine particles and biofilm, whose aggregates eventually sink and may act as a precursor of marine snow [24]. Hence, they may share similarities with acylated polysaccharides (APS), which constitute a major fraction of marine surface water’s high molecular weight dissolved organic matter [57]. Previous analysis of diatom EPS also revealed the presence of heteropolysaccharides, often branched and containing rhamnose, fucose, galactose, glucose, mannose and xylose, which may also be sulfated and contains uronic acids [26].

Diatom-sourced fucoidan was reported as being stable to bacterial degradation, which suggests other biological functions such as protection from pathogens [24]. Fucoidans are of high pharmaceutical interest and were reported for their anti-inflammatory, immunomodulatory and anticoagulant activities [56]. They are also of interest for their anti-proliferative properties on cancer cells and as antiviral agents against many enveloped viruses, including influenza, dengue, human immunodeficiency viruses (HIV), measles and recently SARS-CoV-2 (COVID-19) [58,59,60]. Similar to marennine, it could inhibit the growth of *Vibro alginolyticus*, although the effect was associated with an uncharacterized methanol-soluble fraction extracted from fucoidan, hypothesized as a possible polyphenolic contaminant [61]. Their bioactivity varies according to their structure, and is usually favored by branching, low molecular weight (5–30 kDa), monosaccharides composition and degree of sulfation [55].

NMR spectra showed some similarities in the aliphatic and ring carbons regions between the methanol-soluble fraction and the latest published marennine spectrum [1]. Likewise, retention in membrane of 3.5 kDa MWCO suggests a probable co-extraction during ultrafiltration between 3 and 30 kDa. While the abundance of aliphatic signals could fit a compound composed of monosaccharides comprising methyl and methylene substituents, no anomeric signals were found in the characteristic regions expected in brown algae and diatom fucoidan. Although the FTIR spectrum matched a brown alga fucoidan spectrum and suggested the presence of a sulfated polysaccharide, the NMR results cannot validate this hypothesis. Since fucoidans are defined as fucose-containing sulfated polysaccharides [24,62], confirmation of fucose is required.

The solubility in methanol is also unusual compared to brown algae fucoidans, as they are commonly extracted by alcohol precipitation [55]. Although polysaccharides are usually insoluble in organic solvents, the presence of hydrophobic groups such as *O*-acetyl and *O*-methyl, can also affect their solubility [63], two substituents which are part of fucoidans [55].

Regarding the high resemblance between the polysaccharides fraction and marennine UV spectra, especially for IMn, we speculate that similar compounds remain in the marennine extract. This implies that the methanol-soluble fraction represents only a specific group of other carbohydrates, although its composition remains to be defined. Being polyanionic, compounds similar to fucoidan might be chemically bound to a smaller marennine chromophore, and thus might constitute a major component of the current marennine extracts. This is supported by the comparison of wavelength ratios between different extraction methods, evidences of polysaccharide and aliphatic signals from previous NMR analysis of marennine [1], along with previously reported attributes, such as its polyanionic charge, elevated oxygen content and its high molecular weight, polymeric nature [2].

## 5. Conclusions

Our method provides a straightforward extraction, outperforming ultrafiltration while opening new avenues towards in-line extraction for biotechnology use and further chromophore purification. We demonstrated the potential of low-cost, natural graphite flakes as a stationary phase for marennine pre-concentration, and the effective usage of environmentally suitable solvents and reagents. The process made possible the isolation of a sulfated polysaccharide fraction that requires further characterization. Eventually, methanol and dichloromethane could be replaced by greener solvents. More UV-absorbing compounds are expected to remain in the marennine fraction, either through co-extraction or chemically bound complexes with the chromophore. Hence, we speculate that those might be a major component of marennine extract, and perhaps constitute their active moiety. Still, a thorough characterization of the polysaccharides fraction is necessary to evaluate its purity, bioactivity, molecular weight, monosaccharides composition and degree of sulfation. Confirmation of polysaccharides such as fucans would lead to considering blue water as an important source of bioactive molecules, together with marennine, thus increasing the value of *Haslea ostrearia* cultivation.

## Figures and Tables

**Table 1:** Recovery yields for *Haslea ostrearia* culture in photobioreactors (PBRs), before and after marennine ultrafiltration.

Lot	BW Concentration (mg·L^−1^)	Ultrafiltration Yield (Non-Dialyzed, %)	Ultrafiltration Yield (Reference, %) [10]
1	9.3	54.9	–
2	11.7	55.3	–
3	12.3	59.5	–
Average	11.1 (1.6)	56.6 (2.6)	62.5

**Table 2:** Average crude yield (%) of successive extractions on the same stationary phase, with and without stationary phase pretreatment and reductants in the mobile phase.

Fractions	1	5–10	11–20	Average
Reductants	61 (3)	66 (2)	67 (2)	66 (3)
Blank	47 (3)	66 (2)	67 (2)	64 (5)

**Table 3:** Performance of graphite flakes compared to graphitized carbon black (GCB) (Supelco EnviCarb). AU·g^−1^ refers to absorbance units measured at the visible λ_max_, relative to the stationary phase mass. The letters in parentheses indicate significant differences between treatments.

Stationary Phase	Saturation (AU·g^−1^)	Yield (%)
Graphitized carbon black (GCB)	23.03 (0.97) (a)	64.90 (4.04) (a)
Graphite flakes	15.69 (0.59) (b)	61.97 (1.40) (a)

**Table 4:** Concentration of cations in blue water and through key purification steps (mg·L^−1^).

Purification Step	Yield (%)	Na^+^	K^+^	Mg^2+^	Ca^2+^
Blue water	100	10,095 (56)	348 (8)	1109 (13)	372 (5)
Precipitation	49.5 (1.0)	1609 (32)	5.18 (0.51)	7.10 (0.35)	4.17 (0.08)
Pellet rinse	48.4 (0.3)	1596 (105)	4.30 (1.30)	7.29 (0.05)	3.20 (0.04)
Desalting (SCX)	36.5 (3.9)	51 (89)	0.10 (0.07)	0.24 (0.13)	0.63 (0.48)

**Table 5:** Band assignments for the ATR-FTIR spectra of the contaminant fraction extracted from blue water. Comparison with spectra of fucoidan extracted from brown seaweeds *Padina tetrastromatica* and *Ascophyllum nodosum*.

Experimental Wavelength (cm^−1^)	Fucoidan Wavelength (cm^−1^)	Assignment	Reference
3365 (4)	3448	Hydrogen bonded O–H broad band	[16,17]
2930 (1)	2940	C–H stretching of pyranose ring	[14]
1671 (4)	1688	O–C–O asymmetric stretching	[16]
1441 (0)	1437	O–C–O symmetric stretching	[16,18]
1381 (2)	–	C–H bending	[18]
1195 (7)	1203	C–H deformation of -manuronic residues	[16]
1138 (2)	1140	O=S=O symmetric stretching	[16,17]
844 (0)	844	S=O stretching	[16]
801 (1)	803	Sulphate group absorption band	[16]
723 (1)	724	C–O–S stretching	[16]
601 (1)	601	C=C–H stretching	[16]

**Table 6:** Absorbance ratios of marennine characteristics wavelengths (nm) [2]. Comparison of the methanol-soluble and the marennine fractions from SPE-GCB, ultrafiltrated and dialyzed EMn, and a purified reference.

Sample	Extraction Method	247/322	247/677
Polysaccharides fraction	SPE-GCB	2.53 (0.02)	–
Marennine fraction	SPE-GCB	2.60 (0.04)	5.07 (0.13)
Ultrafiltrated marennine	3–30 kDa	2.86 (0.03)	5.49 (0.07)
Purified reference [2]	3–30 kDa, anion-exchange	2.63	3.50

## Supplementary Materials

The following are available online at https://www.mdpi.com/1660-3397/18/12/653/s1, Figure S1: HSQC ^1^H-^13^C spectrum of the sulfated polysaccharides crude extract in DMSO-d_6_, 25 ${}^{\circ }$C; Figure S2: NMR proton spectrum of the sulfated polysaccharides crude extract in DMSO-d_6_, 25 ${}^{\circ }$C.

## Abbreviations

The following abbreviations are used in this manuscript:

ATR	Attenuated total reflection
BW	Blue water (*Haslea ostrearia* culture supernatant)
DCM	Dichloromethane
DMSO	Dimethyl sulfoxide
EMn	Extracellular marennine
EPS	Exopolysaccharide
FTIR	Fourier-transform infrared spectroscopy
GCB	Graphitized carbon black
HPLC	High-performance liquid chromatography
HSQC	Heteronuclear single quantum correlation
IMn	Intracellular marennine
MP-AES	Microwave plasma-atomic emission spectrometer
MWCO	Molecular weight cutoff
MeOH	Methanol
NMR	Nuclear magnetic resonance spectroscopy
PBR	Photobioreactor
${}_{w}^{s}$pH	Hydroorganic pH value of an electrode calibrated in aqueous buffers
${}_{w}^{w}$pH	Aqueous pH value
PGC	Porous graphitic carbon
PTFE	Polytetrafluoroethylene
SCX	Strong cation exchange
SPE	Solid-phase extraction
TFA	Trifluoroacetic acid
UV–Vis	UV-Visible spectroscopy
