# Supplementary material for: Extraction Improvement of the Bioactive Blue-Green Pigment “Marennine” from Diatom Haslea ostrearia’s Blue Water: A Solid-Phase Method Based on Graphitic Matrices
Source: Mar Drugs. 2020 Dec 18;18(12):653. doi: 10.3390/md18120653 (PMC7767052; doi:10.3390/md18120653)
Supplement: Supplementary file 1 [file marinedrugs-18-00653-s001.pdf]

Supplementary Materials: Extraction improvement of the bioactive blue-green pigment "marennine" from diatom *Haslea ostrearia*'s blue water: a solid-phase method based on graphitic matrices

William Bélanger [1,2], Alexandre A. Arnold[3], François Turcotte[1,2], Richard Saint-Louis[2], Jean-Sébastien Deschênes[4], Bertrand Genard[5], Isabelle Marcotte[3] and Réjean Tremblay[1]

1. Supplementary results

NMR spectra are shown in the following figures. The figure S1 presents the HSQC ^{1}H-^{13}C spectrum of the yellow tinted, sulfated polysaccharides fraction extracted from *Haslea ostrearia*'s blue water, while the proton spectrum appears in figure S2.

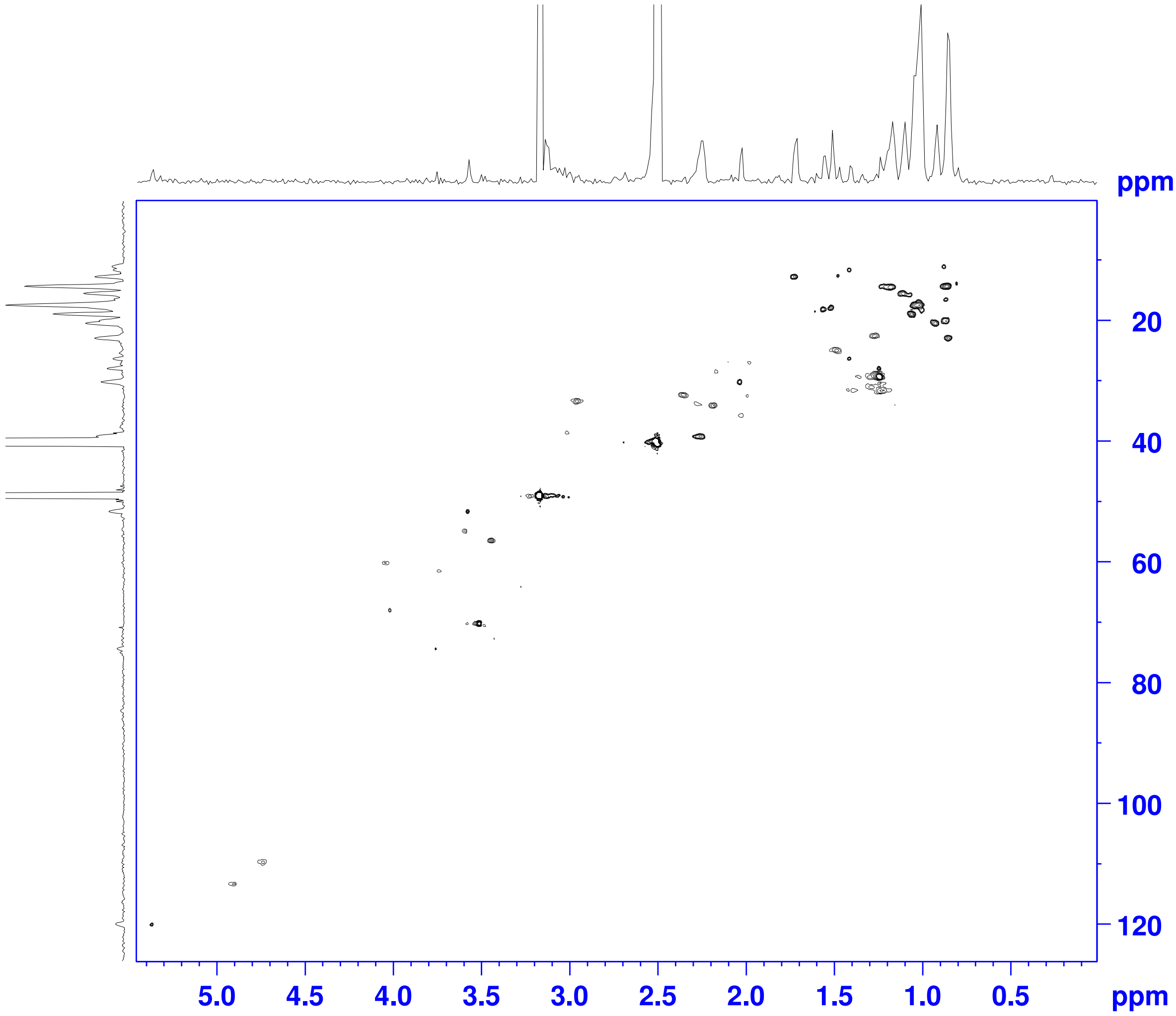

Figure S1. HSQC ^{1}H-^{13}C spectrum of the sulfated polysaccharides crude extract in DMSO-d_6, 25 °C.

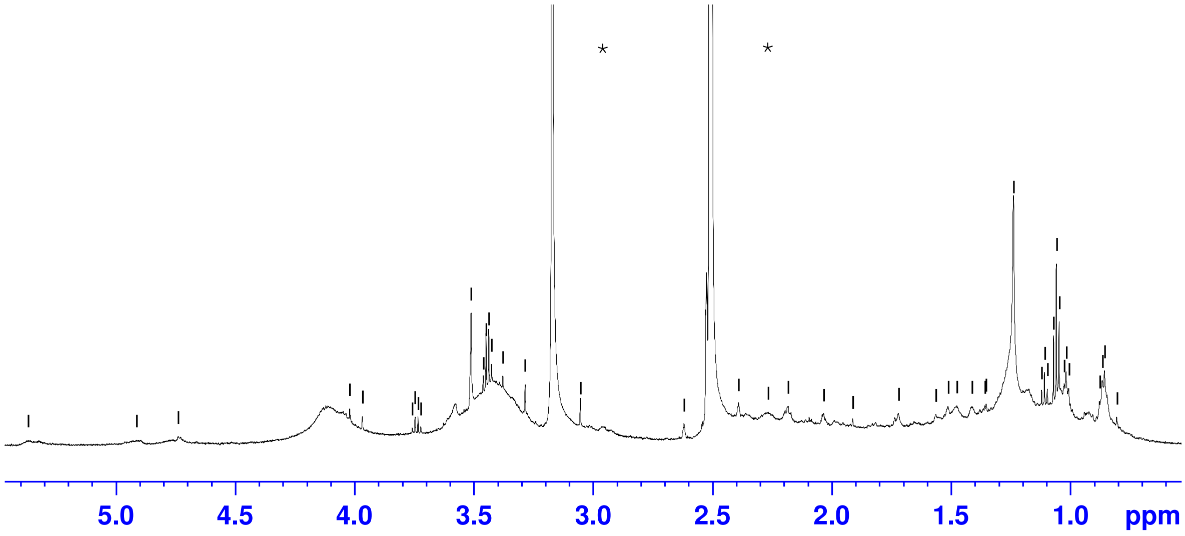

Figure S2. NMR proton spectrum of the sulfated polysaccharides crude extract in DMSO-d_6, 25 °C. Intense signals at 2.5 and 3.2 ppm (*) belong to DMSO and methanol, respectively.

Abbreviations

The following abbreviations are used in this manuscript:

NMR	Nuclear magnetic resonance spectroscopy
